# Noncontact Microembossing Technology for Fabricating Thermoplastic Optical Polymer Microlens Array Sheets

**DOI:** 10.1155/2014/736562

**Published:** 2014-08-04

**Authors:** Xuefeng Chang, Dan Xie, Xiaohong Ge, Hui Li

**Affiliations:** ^1^School of Mechanical and Automotive Engineering, Xiamen University of Technology, Xiamen 361024, China; ^2^Key Laboratory of Precision Actuation and Transmission, Fujian Province University, Xiamen 361024, China; ^3^School of Materials Science and Engineering, Xiamen University of Technology, Xiamen 361024, China

## Abstract

Thermoplastic optical polymers have replaced traditional optical glass for many applications, due to their superior optical performance, mechanical characteristics, low cost, and efficient production process. This paper investigates noncontact microembossing technology used for producing microlens arrays made out of PMMA (polymethyl methacrylate), PS (polyStyrene), and PC (polycarbonate) from a quartz mold, with microhole arrays. An array of planoconvex microlenses are formed because of surface tension caused by applying pressure to the edge of a hole at a certain glass transition temperature. We studied the principle of noncontact microembossing techniques using finite element analysis, in addition to the thermal and mechanical properties of the three polymers. Then, the independently developed hot-embossing equipment was used to fabricate microlens arrays on PMMA, PS, and PC sheets. This is a promising technique for fabricating diverse thermoplastic optical polymer microlens array sheets, with a simple technological process and low production costs.

## 1. Introduction

Thermoplastic optical polymers have replaced traditional optical glass in many applications, owing to their superior optical performance, mechanical characteristics, low cost, and efficient production process. As integral microoptical parts, polymer microlenses and microlens arrays have the advantages of being of small size and low weight, and can be conveniently integrated, thus, playing the critical roles on military affairs, science & technology, and more. The typical production methods include melting photoresist, gray-scale masks, surface micromachining technologies, reactive ion beam etching technologies, photoresist reflow, laser direct-write processing, ion exchange technologies, and microspraying printing [1–11].

The contactless embossing of microlenses, first developed by IMM (Institute of Microtechnology Mainz GmbH) [12], is a novel production technique. The distinct advantage is that it forms in one step, without etching. First, LIGA technology is used to produce a nickel plate with round-hole arrays. Using this plate, the noncontact hot embossing is then performed on a thermoplastic polymer sheet. The polymer forms a microlens when melting, due to the effects of surface tension. The curvature and height of the microlens can be controlled by adjusting the temperature, pressure, and duration of the embossing process. This technology is convenient and flexible, and it provides a quality microlens surface. However, the mold plate production using LIGA technology, which includes using X-ray lithography with deep-etch synchrotron radiation, electroforming molding, and replica molding, is both complex and costly.

In this paper, we demonstrate an alternative method for fabricating thermoplastic polymer microlens arrays using a quartz mold with microhole arrays. The mold was created using traditional photolithography technology. We utilized the three most common thermoplastic optical polymers—PMMA, PS, and PC. By studying the principle of noncontact microembossing techniques using finite element analysis, we decided to employ Ansys to build the model. In addition, we investigated thermal and mechanical properties of the three polymers using a home-made six-axis microtester. Then, we applied independently developed hot-embossing equipment in order to create the microlens arrays using PMMA, PS, and PC [13]. The experimental results indicate that the newly manufactured microlens array has good surface features and high position precision. The noncontact microembossing simplifies the process, due to one-step molding, which is unaffected by the quality of the mold's surface. This demonstrates that noncontact microembossing is of low cost and highly efficient method for fabricating diverse thermoplastic optical polymer microlens array sheets.

## 2. Noncontact Microembossing Technology

### 2.1. Principles of the Fabrication Process

**Figure 1:**
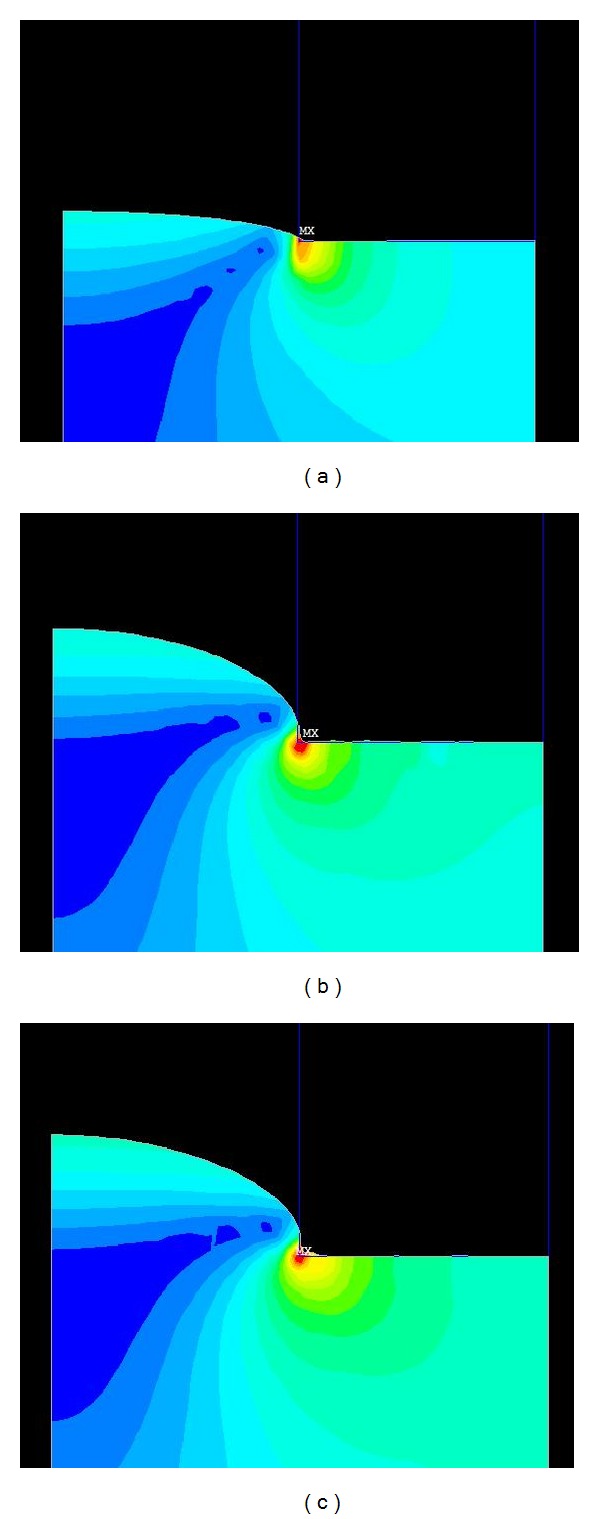
Process of forming a microlens using noncontact microembossing technology.

The prominent characteristic of noncontact microembossing technology is that the planoconvex microlenses can be formed by surface tension, which avoids direct contact between the microlens array surface and the mold's inner surface. The size and position of the microlens are determined by the mold' microhole array. The polymer forms the microlens under the combination of surface tension and pressure, which occurs when the mold plate is heated to above the glass transition temperature.  Figure 1 shows the noncontact microembossing technology process, following three simple steps: (a) the spherical profile of the lens is formed; the height of the sag gradually increases, while the curvature of the radius decreases; (b) the curvature radius has already reached its minimum; (c) the curvature radius ceases to change, but the height of the additional cylinder keeps increasing until releasing the pressure.

### 2.2. Finite Element Analysis

We applied the Moony-Rivlin model to simulate the material characteristics and the stress expressions as follows:

(1) $$\begin{array}{c} \sigma_{i}=\lambda_{i}\frac{\partial U}{\partial \lambda i}\;i=1\sim 3, \end{array}$$

(2) $$\begin{array}{c} U=C_{10}\left(I_{1}-3\right)+C_{01}\left(I_{2}-3\right), \end{array}$$

(3) $$\begin{array}{c} \mu_{0}=\frac{E_{0}}{2\left(1+v\right)}=2\left(C_{10}+C_{01}\right), \end{array}$$

(4) $$\begin{array}{c} C_{01}=0.1C_{10}\sim 0.25C_{10}, \end{array}$$

where *I*_1_ and *I*_2_ are strain invariants; *λ* is the expansivity; *μ*_0_ is the initial shear modulus of the polymer; *E*_0_ is the initial elasticity modulus of the polymer; *ν* is Poisson's ratio of the polymer; and *C*_01_ and *C*_10_ are the related constants of the Moony-Rivlin model [14–16].

In earlier hypotheses of the phenomenological theory, (2) was proposed to express the nonlinear viscoelastic material, wherein the materials were assumed to be incompressible in the Moony-Rivlin model. Even though the strain exceeds 100%, the forecast is still accurate. However, the hardening phenomenon of materials after a large strain cannot be expressed by this model. Since *ν* is equal to 0.5 in terms of the incompressible materials and *E*_0_ is equal to 10 MPa in this experiment, the approximate results can be obtained when *ν* and *E*_0_ are substituted into (3), represented as *C*_01_ ≈ 0.33 MPa, *C*_10_ ≈ 1.33 MPa. In addition, the deformation mechanism during embossing is analyzed by a long-term modulus and static simulation of the material, in which the temperature and pressure during embossing are assumed to be constant.

**Figure 2:**
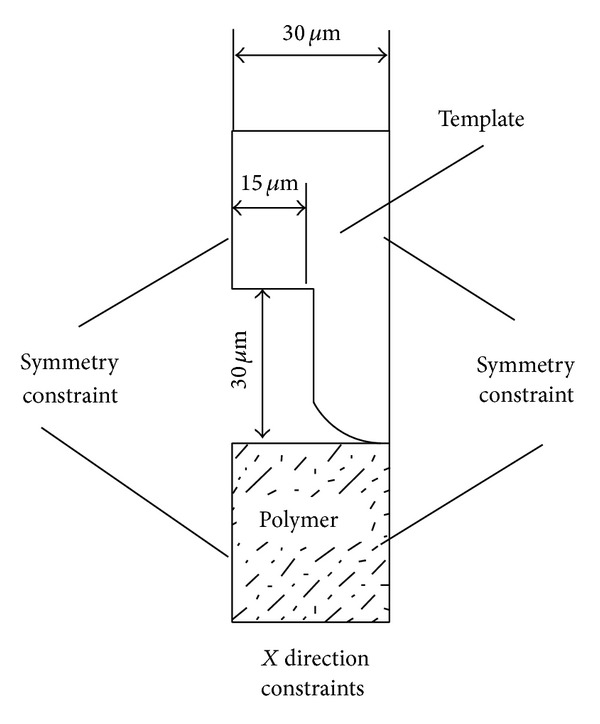
Specifications of the model and boundary conditions.

The aforementioned deformation process of the polymer was simulated using the Ansys. The model was simplified by its symmetry boundaries, because the sectional and geometrical shapes in the vertical direction were repetitive figures.  Figure 2 shows the specifications of the model and its boundary condition. Other assumptions are as follows: (1) the rectangular plane unit and strain simulation are employed to depict the deformation of the polymer; (2) the mold is a rigid body; (3) the polymer deformation can be ignored because it is significantly less than the deformation caused by embossing; and (4) the friction force between the mold surface and the polymer can be ignored. In addition, a serious distortion of the grid during embossing will appear due to the great deformation of the polymer. Thus, it is necessary for the established model to have round corners that prevent the grid from being destroyed or penetrated, which is not in line with the actual conditions.

**Figure 3:**
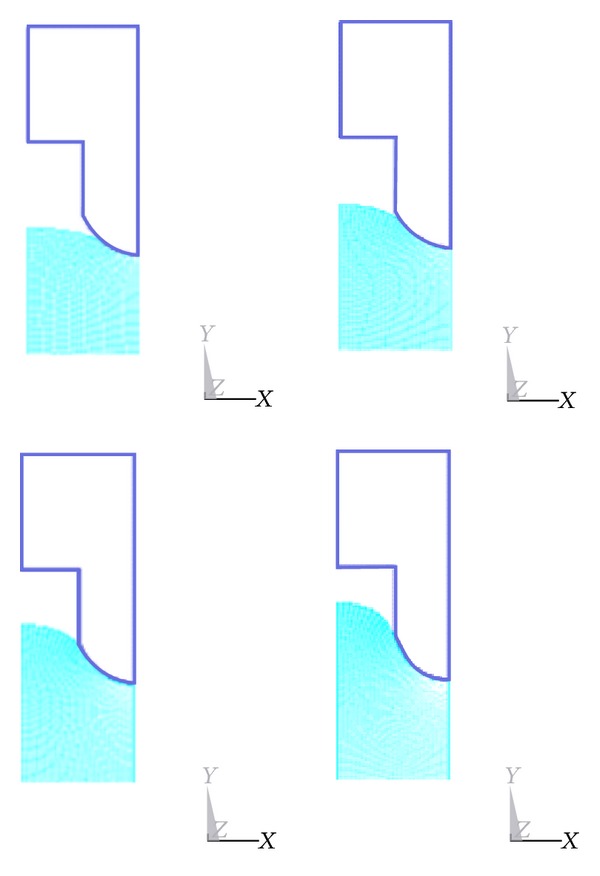
Polymer deformation at the decreased distances of 15, 30, 45, and 60 *μ*m.

**Figure 4:**
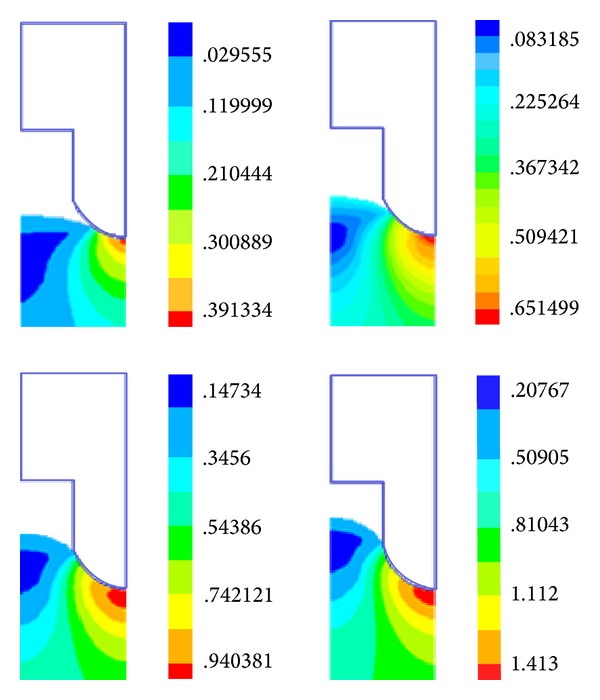
Overall distribution of the strain at the decreased distances of 15, 30, 45, and 60 *μ*m.

In this paper, we employed the updated Lagrange method for nonlinear structural analysis with a large strain. The overall equilibrium equation is the coordinate system for the transition to an equilibrium state of the structure, and the last deformation of this solution is considered to be the reference for the structure solution. Meanwhile, we adopted the Newton-Raphson method for the equilibrium iteration. Furthermore, we employed forced displacement of the rigid body in the mold to simulate the depressing process.  Figure 3 shows the polymer deformation at different decreased distances, whereas Figure 4 shows the overall distribution of the strain at different decreased distances.

### 2.3. Mechanical Properties Investigation of PMMA, PC, and PS

The most salient mechanical properties of thermoplastic polymer are its rubber elasticity and viscoelasticity when in different states, such as the glass state, rubber elastic state, and viscoelastic state. The abovementioned states are closely related to glass transition temperature (*T*_*g*_). In order to determine the appropriate process conditions, including temperature, pressure, and time, we used our home-made six-axis microtester to test the thermomechanical properties for thermoplastic polymers.

**Figure 5:**
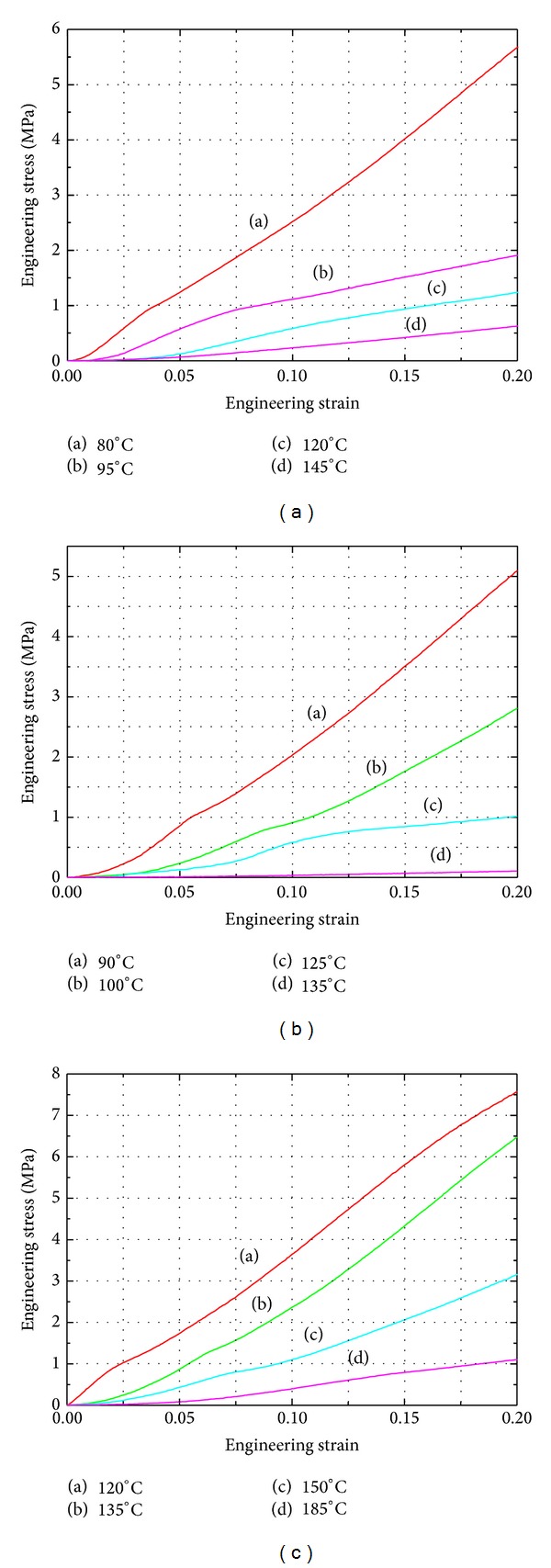
Stress-strain curves of (a) PMMA, (b) PS, and (c) PC at different temperatures with a constant strain state of 0.9*E* − 3 s^−1^.

Figure 5 shows the temperature dependent mechanical behaviors, at a constant stain state, of PMMA and PC by simulating the imprinting process with the temperatures ranging from 80~145°C, 90~135°C, and 120~185°C, respectively. When the temperature is below about 10~15°C of *T*_*g*_, as curve (a) shows, the slope gradient of the stress-strain is the steepest and deformations of the three polymers are difficult because they are in the glass state. When temperature is elevated, the stress levels will decrease as curve (b) shows. Once the temperature is raised above 35~50°C of *T*_*g*_, the three polymers will deform easily, due to low viscosity. It is obvious that polymers under the temperature range of viscoelastic states are the most suitable for embossing.

## 3. Experimental Setup

### 3.1. Mold Production

The mold should meet certain requirements, including stable properties and reusability, as well as the following conditions: (1) extreme hardness, (2) a low coefficient of expansion, and (3) good pick resistance. Our mold material used a square quartz substrate with a thickness of 1 mm and a length of 37 mm.

**Figure 6:**
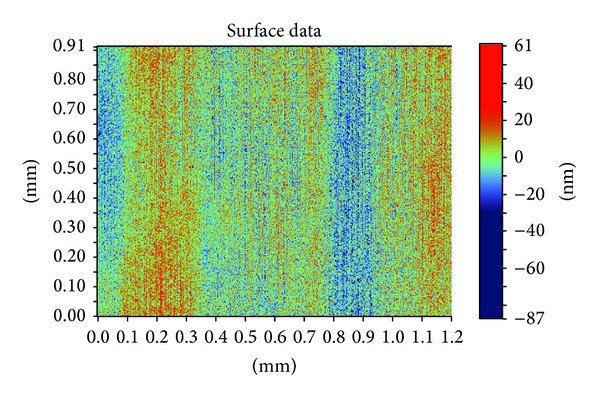
Surface morphology of the Cr layer tested with the optical profiler.

**Figure 7:**
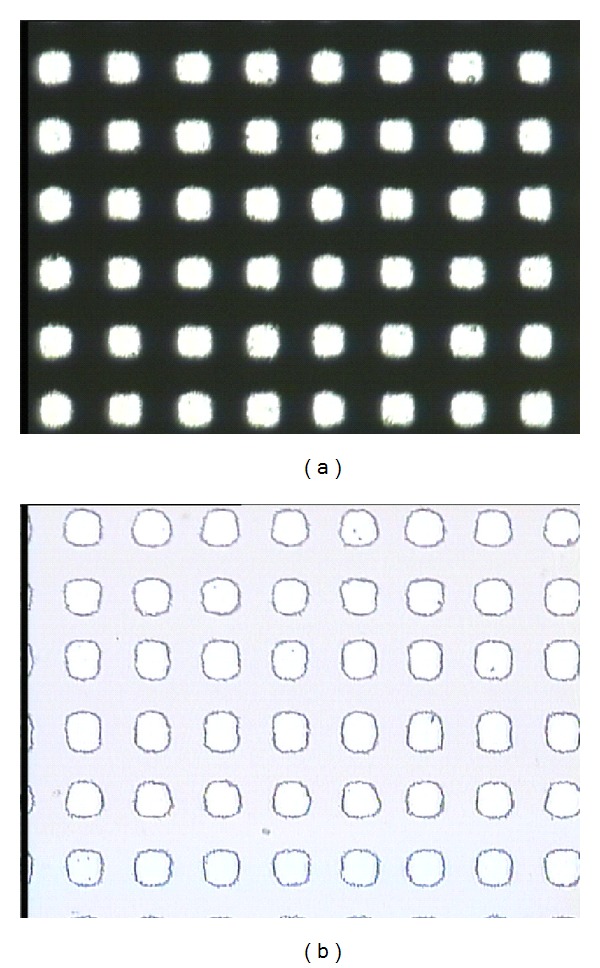
Optical micrograph: (a) photo mask and (b) photoresist pattern after development.

**Figure 8:**
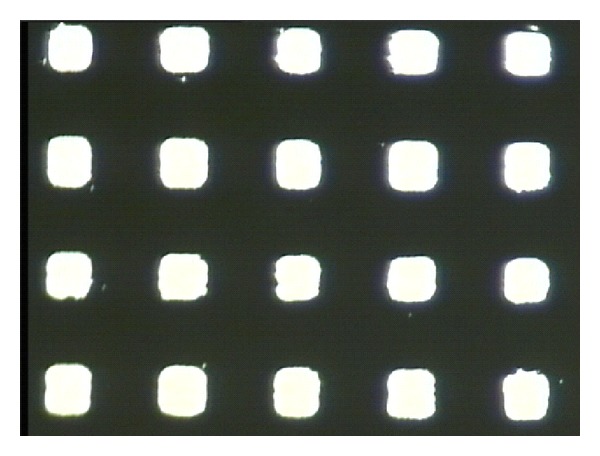
Optical micrograph of the Cr layer after etching.

**Figure 9:**
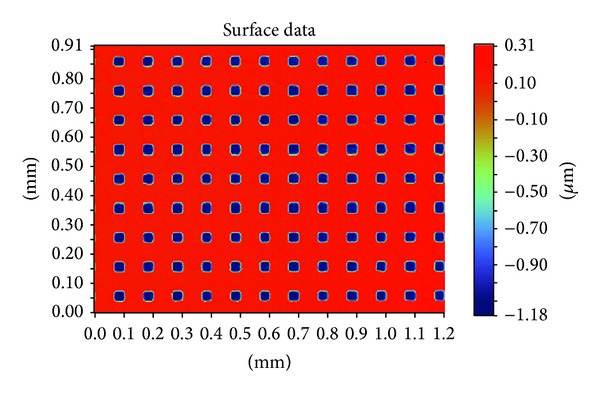
Surface profile of the Cr layer after etching.

**Figure 10:**
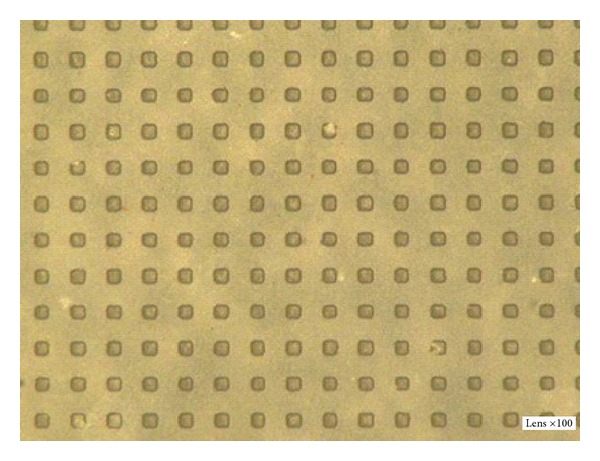
Optical micrograph of the quartz mold by ICP.

**Figure 11:**
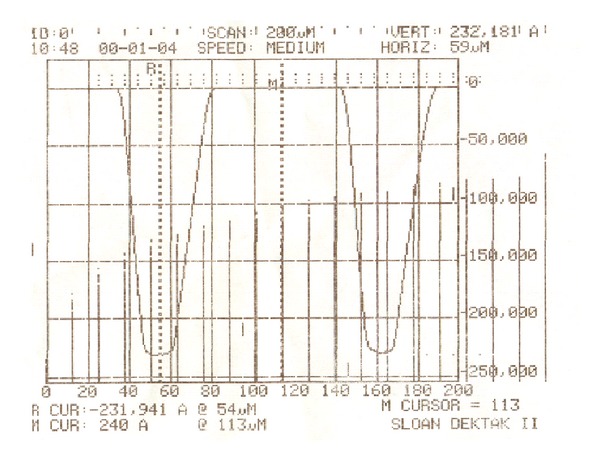
Results of the quartz mold surface with the stylus profiler.

The first step is sputtering, in which the cleaned quartz substrate was placed into the magnetron sputter machine to coat it with a Cr layer. The surface appearance was tested with the optical profiler NT1100 manufactured by Veeco, as shown in Figure 6. The thickness of the Cr film was about 1.2 *μ*m. Then, the quartz substrate was baked for 200~300 seconds at 110°C. The CLARIANT, manufactured positive photoresist AZ6112, was used to spin coat the substrate. A two-step spinning method was adopted; first, the time was set for 6 seconds at 1000 rpm, and then for 30 seconds at 2000 rpm. Next, to ensure good adhesiveness between the resist film and the Cr layer, the quartz substrate was baked again for 200~300 seconds at 110°C using ultraviolet lithography. Karl Suss's system was used to form the microhole patterns. The exposure time was 7 seconds, before the exposed sample was treated in a developing solution for 20~30 seconds.  Figure 7 shows the optical micrograph of photoresist pattern after development. Finally, the post-baking and hard-baking were performed at a temperature of 130°C for 200~300 seconds. The Cr layer was etched using a solution of ammonium cerium nitrate ((NH_4_)_2_Ce(NO_3_)_6_) which contains ammonium cerium nitrate (solid), perchloric acid (liquid), and water. The etch rate was approximately 170 nm/min.  Figure 8 shows the optical micrograph of the Cr layer after etching, whereas Figure 9 shows the surface profile observed by the optical profiler. It can be seen that the Cr film displays good surface smoothness. Then, the quartz microhole mold was produced by ICP etching by using the Cr film as a protective layer.  Figure 10 shows the optical micrograph of the quartz mold.  Figure 11 demonstrates the sweep test results of the mold, as detected by the stylus profiler. The size and the depth of the square hole were 54 *μ*m × 54 *μ*m and 231 *μ*m, respectively. The holes were uniformly distributed and the roughness of the mold surface was good for embossing.

### 3.2. Noncontact Microembossing Process

**Figure 12:**
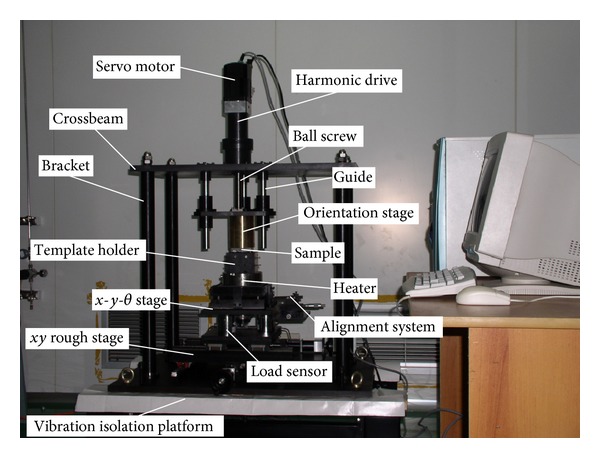
Microcompression molding machine.

Figure 12 demonstrates the self-developed microcompression molding machine. The prototype includes the main following parts: a base, three load sensors, a *Z* movement stage, an alignment system, and a heater. The shock absorption base provides a foundation and isolates vibration from the outside. The three load sensors monitor both the imprint and the separation force. The *Z* movement stage includes a servo motor, a 1 : 150 harmonic drive, and a precision ball screw. The alignment system includes an orientation stage, an *x*-*y* rough stage, a *x*-*y*-*θ* stage, and an optical system. The orientation stage adopts a flexure-based mechanism to guarantee the uniformity of the template and substrate surfaces. The optical system includes coarse and fine pitch gratings, which are adopted on the surfaces of the template and substrate to produce moiré signals, in order to control the macro- and microactuators. This mechanism enables the substrate to move towards the desired position automatically. The surface of the heater is carefully polished to guarantee that the specimens can be loaded by a vacuum chuck.

PMMA, PS, and PC were selected as the samples in this experiment. The sheets (3 mm × 3 mm × 2 mm) that were made out of these materials were ultrasonically cleaned for 2-3 minutes as part of the preparation process. The noncontract microembossing process involved the following. (1) We clamped and leveled the mold. The parallelism of the mold and the polymer sheet was the critical factor for ensuring uniformity in the pattern structure. The orientation stage can guarantee consistency within the template and substrate surfaces. (2)* Preembossing and heating*: we made sure that the mold plate made tight contact with the sample. Then, the sample was heated above its glass transition temperature (*T*_*g*_). The heating temperatures for PMMA, PS, and PC were 130°C, 120°C, and 180°C, respectively. (3)* Embossing*: the compressing process was step by step. The whole operating time was 5 minutes and the embossing depths were 15 *μ*m, 30 *μ*m, 45 *μ*m, and 60 *μ*m, respectively. (4)* Cooling and demolding*: we continued to emboss and lower the temperature to about 60°C simultaneously. After demolding, we had created the polymer microlens array.

**Figure 13:**
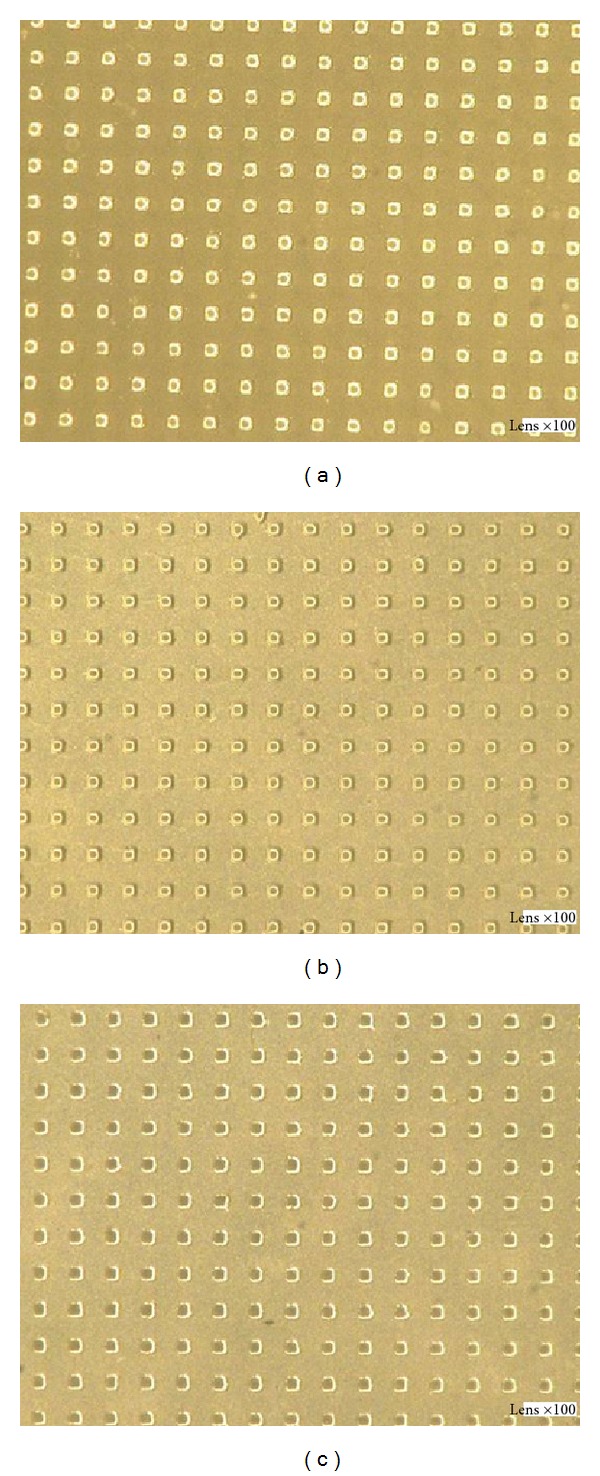
Optical micrographs of focus points of the microlens arrays (×100) (a) PMMA, (b) PS, and (c) PC.

Figure 13 shows the optical micrographs of the focus points of the three kinds of microlens arrays under an optical microscope. The magnification factor is ×100. The images reveal that the pitch and the intensity of the focused light spots are uniform and the view of the focal plane of the microlens array shows uniform arrays among the focal points. In addition, the PS demonstrated the best transmittance when compared with the other two materials. Due to the wide temperature range in its molten state, the PMMA was the most easily controlled material. On the contrary, the molding process of PC is more difficult to control. For square patterns, the noncontact microembossing technology can be employed to easily obtain special-shaped microlenses.

## 4. Conclusion

This paper explores noncontact compression molding technology for fabricating thermoplastic optical microlens arrays. The polymers used were PMMA, PS, and PC and the mold was comprised of a quartz template with microhole arrays. The microlens was formed because of the surface tension of the polymer under the flow dynamic. The results indicated that our one-step molding process was able to hone in on a highly precise position, to create a microlens with a good surface appearance. A simple, precise, and efficient method, noncontact microembossing technology, embodies a great potential for fabricating diverse thermoplastic optical polymer microlens array sheets.
